# A silent strain: the unseen burden of acute respiratory infections in children

**DOI:** 10.1186/s13052-024-01754-2

**Published:** 2024-09-07

**Authors:** Riccardo Boracchini, Benedetta Canova, Pietro Ferrara, Luigi Cantarutti, Carlo Giaquinto, Costanza Di Chiara, Anna Cantarutti

**Affiliations:** 1 Department of Statistics and Quantitative Methods, Division of Biostatistics, Epidemiology and Public Health, Laboratory of Healthcare Research and Pharmacoepidemiology, University of Milan-Bicocca, Via Bicocca Degli Arcimboldi, 8, Milan, 20126 Italy; 2 Center for Public Health Research, University of Milan-Bicocca, Via Cadore 48, Monza, 20900 Italy; 3 Società Servizi Telematici (SoSeTe), Pedianet Project, Via Giacomo Medici 9/A, Padua, 35138 Italy; 4 Department of Women’s and Children’s Health, University of Padova, Via Giustiniani, 3, Padua, 35128 Italy

**Keywords:** Acute respiratory infections, Burden, Pediatricians visits

## Abstract

The significant impact of acute respiratory tract infections on healthcare systems is well-documented, given their contribution to emergency department admissions, hospitalizations, and increased use of antibiotics and other medications. However, further research is needed to understand the burden of acute respiratory tract infections in pediatric community care to develop effective public health interventions and improve child health outcomes. Real-world data were retrieved from Pedianet, an Italian network of over 200 family pediatricians. Acute respiratory tract infection visits were identified and analysed using an infection-duration algorithm to extract individual cases. The number of follow-up visits per 100 cases was calculated to assess the burden on the Italian National Health Service. Comparisons were made overall and stratified by type of acute respiratory tract infections and epidemiological season. A total of 1,402,953 acute respiratory infections-related visits were recorded, with an overall rate of 12 visits per 100 cases. Upper respiratory tract infections had an average of 9 visits per 100 cases. Lower respiratory tract infections exhibited a higher burden, with 29 visits per 100 cases. Pneumonia showed a declining trend in the pre-pandemic era (62 to 48 visits) but rebounded in the post-COVID-19 years (32 to 42 visits). This study underscores the importance of monitoring and managing acute respiratory infections, especially lower respiratory tract infections, in pediatric care.

**Supplementary Information:**

The online version contains supplementary material available at 10.1186/s13052-024-01754-2.

## Background

Acute Respiratory Infections (ARIs) significantly impact children’s health, and place a considerable burden on the Italian National Health Service (SSN) [1]. This retrospective, observational study quantified the ARIs burden by analysing real-world, community-based outpatient visit data.

## Methods

Data on ARIs, from September 23, 2010, to April 30, 2024, were retrieved from the Pedianet database, a network encompassing over 200 Italian family pediatricians (FPs). Pedianet (http://www.pedianet.it) captures real-world outpatient data about children, including demographics, health status, medications, and symptoms [2]. ARIs were identified using International Classification of Diseases, Ninth revision, Clinical Modification (ICD-9-CM) codes and a free-text field validated by a clinical data manager (Table S1). Data generated during routine patient care were collected and handled anonymously, in compliance with Italian regulations, and stored under a unique numerical identifier.

A time-based definition was employed to distinguish distinct ARI episodes, incorporating a minimum infection duration criterion to exclude follow-up visits (Figure S1) [3–5]:

i. Intra-diagnoses: at least 30-day between two different pneumonia episodes and 15-day between two different other not-pneumonia ARI diagnoses;
ii. Intra-respiratory tract categories: at least 15-day between two different episodes of Upper Respiratory Tract Infections (URTIs) and two different episodes of Lower Respiratory Tract Infections (LRTIs);
iii. Inter-respiratory tract categories: at least 15-day between a first episode of LRTI and a subsequent episode of URTI and vice versa.

Descriptive statistics, including frequencies and percentages, were used to characterize the acute cases and their associated visits.

The number of follow-up visits per 100 cases was calculated to assess the burden of infection management on the Italian SSN. This indicator was analysed according to the epidemiological season, defined as the timeframe from 23/09/20xx to 22/09/20xx + 1. All the analyses were stratified according to the most frequent ARIs. Statistical analyses were performed using SAS software version 9.4. We followed the Strengthening of the Reporting of Observational Studies in Epidemiology (STROBE) guidelines.

## Results

**Fig. 1:**
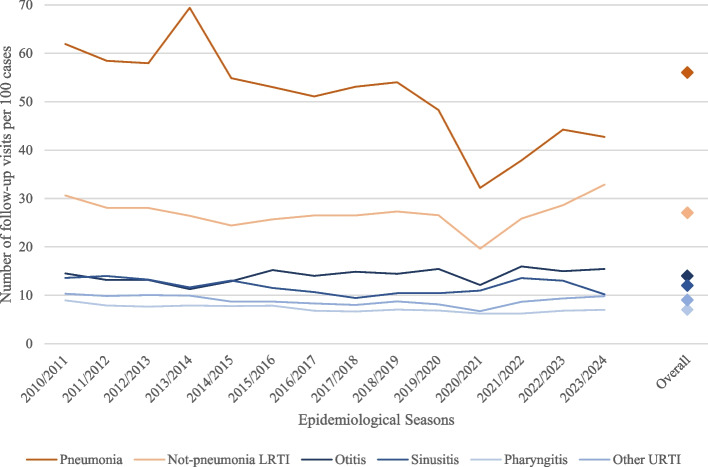
Number of follow-up visits for 100 cases during epidemiological seasons 2010/2011 – 2023/2024 by ARIs. Abbreviations: ARI, Acute Respiratory Infection; URTI, Upper Respiratory Tract Infection; LRTI, Lower Respiratory Tract Infection

A total of 356,699 children entered the study, accounting for 1,402,953 ARI-related visits. According to our time-based definition, we identified 150,812 (10.75%) follow-up visits. The percentage of follow-up visits varied by infection site, ranging from 6.94% for pharyngitis to 35.65% for pneumonias. URTIs displayed an average of 9 follow-up visits per 100 infections, with individual visit frequencies varying across specific URTI subtypes (7, 12, 14, 9 visits for 100 pharyngitis, sinusitis, otitis, other URTIs, respectively). LRTIs exhibited a higher average visit frequency of 29 visits per 100 infections, with pneumonia accounting for 55 visits and other not-pneumonia LRTIs 27 visits (Table 1, Fig. 1).

**Table 1:** Number of cases, visits, and follow-up visits per 100 cases by infection site

ARI	Cases	Visits	Follow-up visits per 100 cases
Overall – N (%)	1,252,141 (100)	1,402,953 (100)	12
URTIs – N (%)	1,065,264 (85.08)	1,162,051 (82.83)	9
Pharyngitis	318,475 (25.43)	342,198 (24.39)	7
Sinusitis	20,257 (1.62)	22,704 (1.62)	12
Otitis	70,947 (5.67)	81,219 (5.79)	14
Other URTIs	655,585 (52.36)	715,930 (51.03)	9
LRTIs – N (%)	186,877 (14.92)	240,902 (17.17)	29
Pneumonia	10,313 (0.82)	16,025 (1.14)	55
Not-pneumonia LRTIs	176,564 (14.10)	224,877 (16.03)	27

*Abbreviations*: *ARI* Acute Respiratory Infection, *URTI* Upper Respiratory Tract Infection, *LRTI* Lower Respiratory Tract Infection

Focusing on the trend over the epidemiological seasons, as expected, pneumonia showed the highest-burden decrease, dropping from 62 to 43 visits for 100 cases, with the lowest value recorded in the COVID-19 year (i.e. 2020/2021, 32 visits). Other not-pneumonia LRTIs demonstrated an increase in the post-pandemic years, reaching 33 visits per 100 cases in the 2023/2024 season. The burden of other upper and lower ARIs appeared consistent over time (Fig. 1).

## Discussion

This 13-year study investigated the burden of ARIs on the Italian SSN by evaluating follow-up visit frequencies per 100 cases using real-world data from outpatient pediatric practices. Using novel time-based definition of ARI episodes, we identified the single acute ARI episodes and compared them between diagnoses and over time. Our results provide significant insights into the burden of ARIs within primary pediatric care in Italy, consistent with the international literature [6–8]. The study findings provide information on the utilization of healthcare visits associated with specific sub-diagnoses, particularly for LRTIs, in children. In addition to clinical adverse outcomes, from a healthcare perspective, managing pediatric ARIs is complex and highly resource-consuming, involving frequent access to health services, diagnostic procedures, and drug prescriptions [1, 7, 9].

## Conclusions

Our study demonstrates the substantial burden of ARIs on pediatric primary care, as evidenced by the high rate of follow-up visits highlighting the need for tailored management strategies. By leveraging real-world data from a large network of FPs, we emphasize the importance of ongoing surveillance to inform resource allocation and public health interventions.

## Supplementary Information

Supplementary Material 1.

## Data Availability

Data may be obtained from a third party and are not publicly available.

## Abbreviations

ARI: Acute respiratory infection
SSN: National health service
FP: Family pediatricians
ICD-9-CM: International Classification of Diseases, Ninth revision, Clinical Modification
LRTI: Lower Respiratory Tract Infection
URTI: Upper Respiratory Tract Infection
STROBE: Strengthening of the reporting of observational studies
