# Supplementary material for: A silent strain: the unseen burden of acute respiratory infections in children
Source: Ital J Pediatr. 2024 Sep 7;50:167. doi: 10.1186/s13052-024-01754-2 (PMC11380415; doi:10.1186/s13052-024-01754-2)
Supplement: Supplementary file 1 — Supplementary Material 1. [file 13052_2024_1754_MOESM1_ESM.pdf]

A Silent Strain: The Unseen Burden of Acute Respiratory Infections in Children

Correspondence: riccardo.boracchini@unimib.it, +39 345 3242013 (phone number)

Additional file 1

Table S1. Acute Respiratory Infections classifications according to the International Classification of Diseases, Ninth Revision, Clinical Modification (ICD-9-CM).

Figure S1. Time-based definition for Acute Respiratory Infections

Table S1. Acute Respiratory Infections classifications according to the ICD-9-CM.

Diagnosis	ICD-9-CM code	Free-text string
Lower Respiratory Tract Infections		
Pneumonia	481 Pneumococcal pneumonia [Streptococcus pneumoniae pneumonia]<br>482 Other bacterial pneumonia<br>483 Pneumonia due to other specified organism<br>484 Pneumonia in infectious diseases classified elsewhere<br>485 Bronchopneumonia, organism unspecified<br>486 Pneumonia, organism unspecified	"*polmonit*", "*Polmonit*", "*POLMONIT*", "* bp*", "* BP*", "*bmp*", "* BMP*", "* bpn*", "* BPN*"
Other	466 Bronchiolitis<br>490 Acute bronchitis<br>491 Chronic bronchitis<br>519.11, 786.07 Wheezing episode	"Bronchit*", "BRONCHIT*", "bronchit*", "*Bronchit*", "*BRONCHIT*", "*bronchit*", "Bronchiol*", "BRONCHIOL*", "bronchiol*", "*Bronchiol*", "*BRONCHIOL*", "*bronchiol*", "*ezing*", "*EZING*", "*oncospasm*", "*ONCOSPASM*"
Upper Respiratory Tract Infections		
Pharyngitis	034 Streptococcal sore throat and scarlet fever<br>462 Acute pharyngitis<br>463 Acute tonsillitis	"FARINGIT*", "Faringit*", "faringit*", "* faringit*", "* Faringit*", "* FARINGIT*", "*onsillit*", "*ONSILLIT*", "* FT*", "* Ft*", "* ft*"
Sinusitis	461 Acute sinusitis<br>473 Chronic sinusitis	*sinusit*, *Sinusit*, *SINUSIT*, sinusit*, *Sinusit
Suppurative otitis media	382 Suppurative and unspecified otitis media	"otit*", "Otit*", "OTIT*", "* otit*", "* Otit*", "* OTIT*", "OMA*", "oma*", "* OMA*", "* oma*", "OME*", "ome*", "* OME*", "* ome*"
Non-suppurative otitis media	381 Non-suppurative otitis media and Eustachian tube disorders	
Other	464 Laryngitis – tracheitis<br>460.x Upper respiratory tract infection	"*inofaring*", "*INOFARING*", "Rinit*", "RINIT*", "rinit*", "* RINIT*", "* Rinit*", "* rinit*", "*affred*", "*AFFRED*", "*racheit*", "*RACHEIT*", "laringit*", "Laringit*", "LARINGIT*", "* laringit*", "* Laringit*", "* LARINGIT*", "* respiratorie*", "* RESPIRATORIE*", "* Respiratorie*", "URI *", "uri *", "* URI *"<br>, "* uri *", "ivas *", "IVAS *", "* ivas *", "* IVAS *", "Influenza*", "INFLUENZA*", "influenza*", "* Influenza*", "* INFLUENZA*", "* influenza*"

Figure S1. Time-based definition for Acute Respiratory Infections

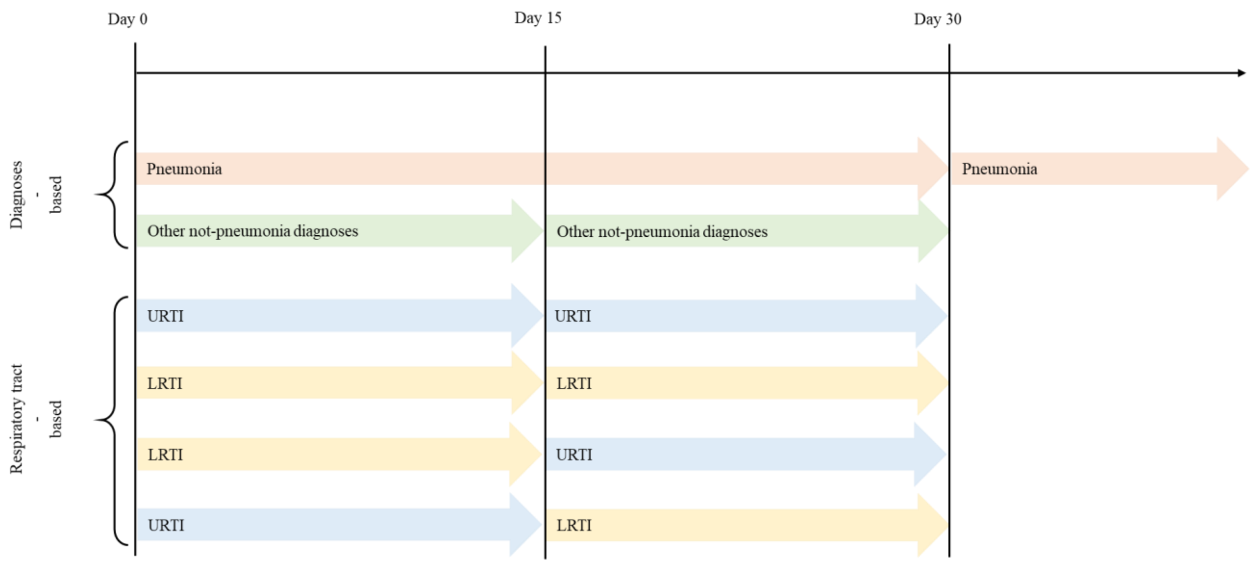

Abbreviations: URTI, Upper Respiratory Tract infection; LRTI, Lower Respiratory Tract Infection
